# *Drosophila* Wing Integration and Modularity: A Multi-Level Approach to Understand the History of Morphological Structures

**DOI:** 10.3390/biology11040567

**Published:** 2022-04-08

**Authors:** Hugo A. Benítez, Thomas A. Püschel, Manuel J. Suazo

**Affiliations:** 1 Laboratorio de Ecología y Morfometría Evolutiva, Centro de Investigación de Estudios Avanzados del Maule, Universidad Católica del Maule, Talca 3466706, Chile; 2 Centro de Investigación en Recursos Naturales y Sustentabilidad (CIRENYS), Universidad Bernardo O’Higgins, Avenida Viel 1497, Santiago 8370993, Chile; 3 Ecology and Evolutionary Biology Division, School of Biological Sciences, University of Reading, Reading RG6 6AH, UK; t.a.puschel@reading.ac.uk; 4 Institute of Human Sciences, School of Anthropology and Museum Ethnography, University of Oxford, Oxford OX1 2JD, UK; 5 Instituto de Alta Investigación, Universidad de Tarapacá, Casilla 7D, Arica 1000000, Chile; suazo.mj@gmail.com

**Keywords:** geometric morphometrics, static integration, developmental integration, evolutionary integration, *Drosophila*, fluctuating asymmetry

## Abstract

**Simple Summary:**

The diverse components of any morphological structure are integrated with respect to each other since they have developed, functioned, and evolved together, a phenomenon known as integration. However, this integration is not absolute but organized in units (i.e., modules) that are relatively independent while participating to generate a structure that acts as a functional whole. Even though most of the studies on modularity and integration have focused on variation among individuals within populations, there are more levels of variation that exhibit modularity and integration, deriving from distinct sources such as genetic variation, phenotypic plasticity, fluctuating asymmetry, evolutionary change, among others. Consequently, the present study focused on analysing the integration and modularity of the wing shape of some of the best-known model organisms, i.e., the genus *Drosophila*, at the static, developmental, and evolutionary levels to acquire a better insight about how modularity and integration act at different analytical levels. The strong integration and overall similarities observed in the variation pattern at multiple levels suggest a shared mechanism underlying the observed variation in *Drosophila*’s wing shape and added a new piece of evidence of stasis in the evolutionary history of *Drosophila* wing.

**Abstract:**

Static, developmental, and evolutionary variation are different sources of morphological variation which can be quantified using morphometrics tools. In the present study we have carried out a comparative multiple level study of integration (i.e., static, developmental, and evolutionary) to acquire insight about the relationships that exist between different integration levels, as well as to better understand their involvement in the evolutionary processes related to the diversification of *Drosophila*’s wing shape. This approach was applied to analyse wing evolution in 59 species across the whole genus in a large dataset (~10,000 wings were studied). Static integration was analysed using principal component analysis, thus providing an integration measurement for overall wing shape. Developmental integration was studied between wing parts by using a partial least squares method between the anterior and posterior compartments of the wing. Evolutionary integration was analysed using independent contrasts. The present results show that all *Drosophila* species exhibit strong morphological integration at different levels. The strong integration and overall similarities observed at multiple integration levels suggest a shared mechanism underlying this variation, which could result as consequence of genetic drift acting on the wing shape of *Drosophila*.

## 1. Introduction

Understanding integration and modularity is essential to acquire insight about the evolution of shape since the coherence of organismal parts depends on their structure, function, and developmental origin [1,2,3]. Morphological integration has been defined as the cohesion among traits that results from the interactions of the biological processes generating the observed phenotypes [3]. The integration of a morphological structure means that the different parts composing this specific structure covary with respect to each other. In an extreme scenario it means that all parts are perfectly correlated, thus the variation of the relative positions of, e.g., landmarks in any sub-set region would be enough to perfectly predict the variation of the relative positions of the remaining landmarks in the rest of the structure. If relations are linear, this also implies that all variation is confined to a single dimension of shape space [4].

Modularity exists if integration is focused within certain regions or parts of a structure (i.e., the modules), but is comparatively weak between these modules [3,5,6]. Morphological modularity consequently implies that integration in a structure is divided into compartments, with strong within-module and weak between-module integration. Modularity, due to the weak integration among modules, can then alleviate the effects of restraints that would apply if variation were entirely integrated [3,5,6,7,8,9].

Analyses of morphological integration and modularity have been carried out with geometric morphometric techniques, mainly because these methods provide diverse statistical tools to address specific biological questions regarding modularity and integration [8,9,10,11,12,13]. The core of geometric morphometrics is based on the combination of multivariate statistics and geometry, thus ensuring that the shape of a structure is consistently described [14,15,16].

Klingenberg [4] indicates that mainly in empirical studies using geometric morphometrics seven levels of morphological integration are well defined. Five of these levels are defined in a static context, which means starting from a single population at a particular ontogenetic stage: e.g., static, functional-environmental, developmental and genetic integration and the other levels are related to analysis of several growth stages or species: ontogenetic and evolutionary integration [3,4,17,18,19,20,21,22,23,24]. Consequently, understanding the different levels of integration allow the comparison between patterns of covariation, thus acquiring insight about evolutionary and developmental processes generating these patterns [2,17,25,26].

Due to their relative structural simplicity, *Drosophila* wings provide an outstanding system to analyse morphological variation, because well-defined landmarks can be established on the wing vein intersections making them very suitable for morphometrics [27]. For instance, authors such as Klingenberg and Zaklan [7] and Klingenberg [28] have confirmed that *Drosophila melanogaster* wings are good models for integration studies. They conclude that the *Drosophila* wing behaves as complete integrated unit. Nevertheless, currently there are no morphometric studies of integration at different levels on *Drosophila* or studies of morphological integration in comparative contexts, which are required to confirm if the observed pattern of integration has evolved across the genus *Drosophila*. Therefore, we have carried out a comparative multiple level study of integration (i.e., static, developmental, and evolutionary) to acquire insight about the relationships between different integration levels, and how they are involved in the evolutionary processes related to the diversification of the *Drosophila* wing shape. This study would not only provide significant information about the integration of a commonly used model structure (i.e., *Drosophila*’s wing) but also can be regarded as an example of how to analyse different integration levels using geometric morphometric data.

## 2. Materials and Methods

### 2.1. Samples and Shape Analyses

**Figure 1:**
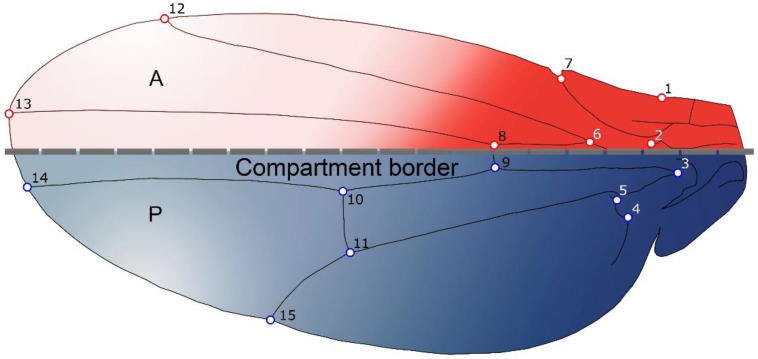
Dorsal view of *Drosophila* wing morphology showing the 15 landmarks used to characterize its shape. The dotted line indicates the boundary between the anterior (red) and posterior (blue) compartments.

All the analyses were conducted with 59 species belonging to the genus *Drosophila*. 50 taxa were provided by the *Drosophila* Species Stock Centre at the University of California, San Diego, which currently maintains a living collection of over 250 *Drosophila* species represented by approximately 1600 stocks that are used by biological researchers focusing on questions in evolution, ecology, developmental biology, physiology, neurobiology, comparative genomics, and immunology. The other nine taxa correspond to specimens used in Klingenberg and Gidaszewski [29]. Approximately 50 wings for each sex were processed for every species (removed and mounted on microscope slides) to carry out the morphometrics analyses described below. All the wings were digitized by collecting 15 landmarks in the dorsal view of the wing (Figure 1). These landmarks were located at vein intersections with other veins and margins, so that all the coordinates were easily located and clearly homologous.

### 2.2. Measurement Error

Measurement error (ME) is always undesirable but also inevitable. It therefore requires to be minimised so that it does not interfere with the most subtle effects of interest under study. In order to calculate the measurement error, the left and right wings of four individuals belonging to five species across the genus (40 wings in total) were digitised twice [30] and a Procrustes ANOVA was calculated. The Mean square (MS) relate to the individual effect and were used as an estimator of an individual’s variation, while the MS related to the interaction (individual*side) left and right side were used as an estimator of fluctuating asymmetry (FA) and compared with the digitized Error 1 to assess measurement error.

### 2.3. Comparative Analysis

**Figure 2:**
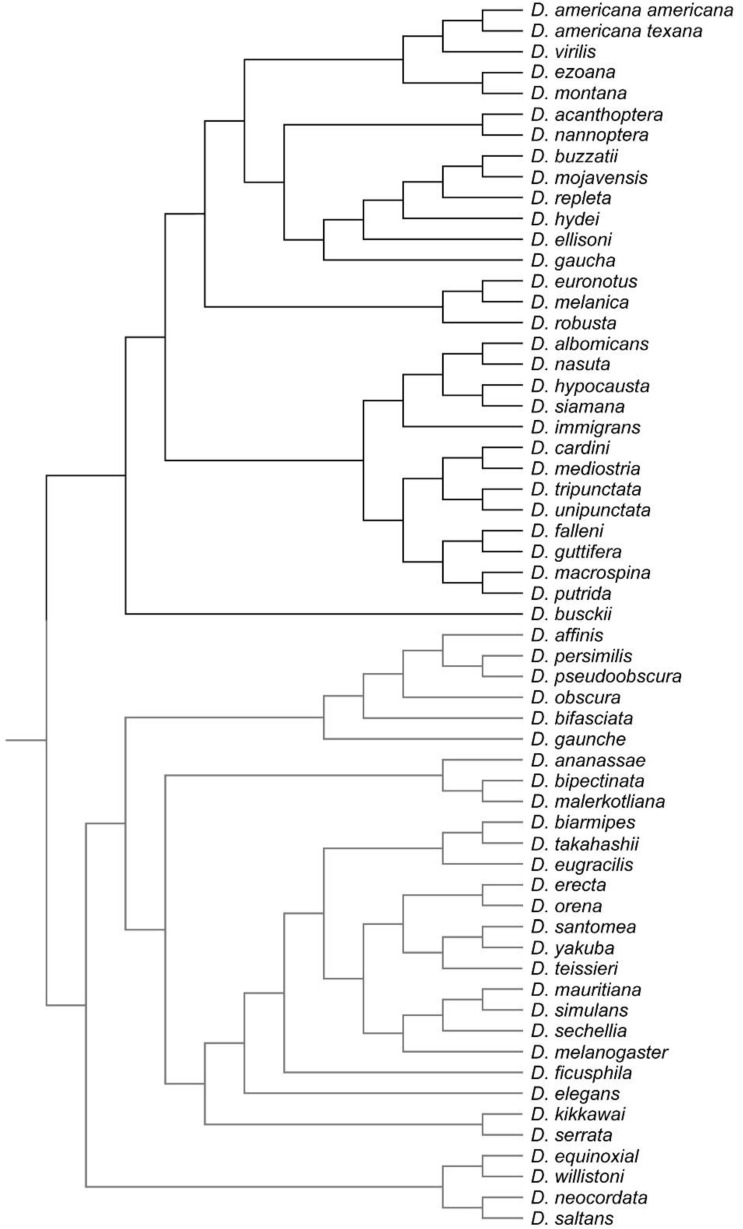
Reconstructed phylogeny using 59 species of *Drosophila* based on the phylogeny from Russo et al. [31] and Van der linde et al. [32] (see text for details).

The phylogenetic trees of Russo et al. [31] and Van der Linde et al. [32] were used as references to build a composite phylogeny that was used in all our comparative analyses. This phylogeny comprises the 59 taxa for which we have shape data (Figure 2) and it was built in Mesquite 3.10 [33]. The phylogenetic relationships between 55 species were established based on Russo et al. [31], while the positions of *D. acanthoptera*, *D. fallenii* and *D. macrospina* were based on Van der Linde et al. [32]. The resulting reconstructed phylogeny was used to map the shape data by square-change parsimony [29,34] and to compute independent contrasts [35]. A permutation approach using 10,000 random permutations was performed to assess phylogenetic signal in the morphometric data. All the analyses were performed in MorphoJ 1.06e [36].

### 2.4. Multi-Levels Approach

Three levels of variation were examined in the present study:

- Static Level: This is basically the level of variation among individuals in a consistent sample, where all individuals belong to the same species and ontogenetic phase [4]. For this level, integration and modularity patterns were studied by examining the variation patterns derived from the pooled within species and sex covariance matrix of wing shape.
- Developmental Level: This level arises from the interactions between developmental processes that generate different traits, and hence produce covariation between them [4]. Covariation arises as result of the processes that generated the morphological structures under study, and it is therefore possible, within certain boundaries, to use morphological covariation to infer how the traits interact developmentally. The study of FA is an effective manner to remove genetic and environmental variation among individuals [3], as the left and right sides of an structure share the same genome and almost the same environmental circumstances, hence the differences between the sides can be assumed to be derived from random fluctuations during the developmental process [3,32,37,38]. Therefore, we used the covariance matrix of fluctuating asymmetry to analyse developmental level integration (i.e., this will allow us to study directly the intrinsic, developmental component of integration and modularity). It is important to keep in mind that the calculation of the FA is provided by the ANOVAs for shape considering individual and side effects, and the interaction between them. The MS related to the individual effect was used as an estimator of individual variation, and the MS related to the interaction (individual x side) as an estimator of FA.
- Evolutionary Level: Covariation among evolutionary changes in different features, arise from several processes including drift, mutation, selection and gene flow [4]. To study evolutionary integration and modularity a comparative approach is required to consider the phylogenetic structure of the data. Consequently, morphological integration and modularity across *Drosophila* species can be assessed by studying the relations between shape features and the evolutionary changes along the branches of the phylogeny.

### 2.5. Integration

To examine the levels of overall morphological integration at multiple levels, a principal component analysis (PCA) was carried out; this technique focuses on the dimensionality of variation, thus providing an integration measurement for an overall structure. The PCA identified the first few eigenvectors as the main elements of shape variation and the resultant eigenvectors associated with each PC represent the relative amount of variation [39]. In order to estimate the overall levels of integration, the procedures described in Gómez et al. [40] were applied to calculate the total of eigenvalues. The calculation of the total variance of shape was obtained by summing the variances of all Procrustes coordinates for each species. This value is known as eigenvalue variance scaled by total variance and number of variables.

To test the morphological integration between the known developmental wing compartments (i.e., anterior and the posterior) [7] (Figure 1), the patterns of covariation were studied. They were analysed by means of a partial least squares (PLS) analysis [37,38]. PLS axes provide new shape variables that maximise the covariance between the landmark configurations of the different parts, and therefore can be interpreted as the main features of integration between them [41]. A permutation approach was also used to test for significance using 10,000 randomization rounds.

### 2.6. Modularity

For the analysis of modularity, the null hypothesis tested in the present study was taken from previous studies about *Drosophila melanogaster* wing integration that indicated that the anterior and posterior parts of the wings are separate developmental units that vary independently. It is important to notice that Klingenberg and Zaklan [7] reject their hypothesis of modularity for *D. melanogaster* although this was never tested for other species of the genus [7,28]. In geometric morphometrics modularity hypotheses have been examined by testing the strength of covariation between the configuration of landmarks into sub-partitions representing the hypothesized modules and alternative sub-partitions into random subsets of landmarks [24]. Here we analysed this hypothesis using the landmarks 1,2,6,7,8,12 and 13 for the anterior compartment and 3, 4, 5, 9, 10, 11, 14 and 15 for the posterior compartment (Figure 1) for all taxa. The effect size of modularity was analysed by using the RV coefficient implemented in MorphoJ and by means of the CR coefficient using the R package “geomorph” via the gmShiny interface [42]. We included the CR coefficient, since Adams [10] argue that the RV coefficient is sensitive towards differences in the sample size, which would hinder a comparison between different data sets.

### 2.7. Allometry

Allometry is a key factor for integration and modularity when shape variation is related with size variation [2,28,43]. Since the relationship between shape and size is linear or almost linear, the allometric effects of size variation are mostly concentrated in a single dimension of the shape tangent space. Furthermore, due to the fact that allometric variation can account for a sizeable proportion of total shape variation, that allometry can have a considerable role in the overall integration of shape [24]. Allometric effects were assessed for the different levels described above (Section 2.4) by performing a multivariate regression of the centroid size on the wing shape [44,45].

### 2.8. Comparison within Levels

To compare the different levels, a series of visualizations showing morphological changes associated with PC and PLS axes were generated. Due to the fact that covariation patterns were characterized as covariance matrices of shape coordinates, their differences between covariance matrices were assessed by using a matrix correlation approach [24]. Furthermore, to quantitatively compare the results, a series of angular comparisons between the landmark vectors were used [24] as a direct way to measure the similarity of the angles between two vectors in the shape tangent space [5,18,46,47].

## 3. Results

The Procrustes ANOVA applied to assess measurement error showed that the mean square for individual variation exceeded ME; therefore, we can consider it to be negligible (Table 1).

### 3.1. Static Level

**Figure 3:**
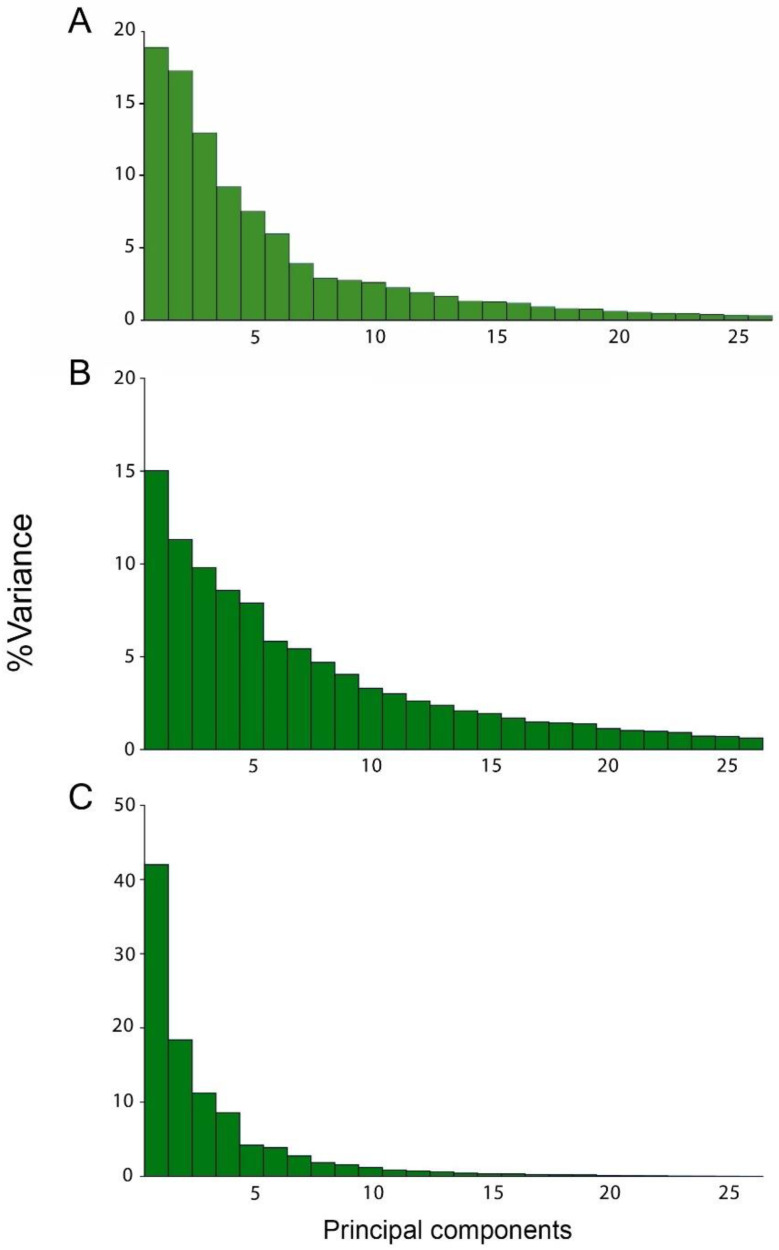
Percentages of total shape variation and overall structure integration by principal component (PCA) using covariance matrices (CM) of: (**A**) CM pooled within-species (**B**) CM of fluctuating asymmetry, (**C**) CM of shape independent contrasts.

The PCA carried out to analyse the static integration of wing shape within-species showed that the first three shape dimensions, accounted for about half of the shape variation (Figure 3A, Table 2). The analysis showed a moderate static integration for the wing shape (i.e., PC1: 18.92%, PC2: 17.3% and PC3: 13.004%). The overall level of integration accounted for 0.07079.

Static integration across species had a total percentage of variation in their first three PC’s ranging from 52% to 86% depending on the *Drosophila* species. Most of the species independently showed very high levels of static integration (Percentage of variation values by species are provided in Supplementary Table S1).

The overall strength of association between compartments indicated a significant relationship between A/P compartments (RV: 0.4198; *p* < 0.001). Most of the species separately showed relatively high level of integration between wing compartments (Supplementary Table S2).

The PLS analysis between A/P wing compartments showed that the first two pairs of PLS axes accounted for the 70.69% (PLS1: 43.03% and PLS2: 27.66%) of the total squared covariance between blocks.

**Figure 4:**
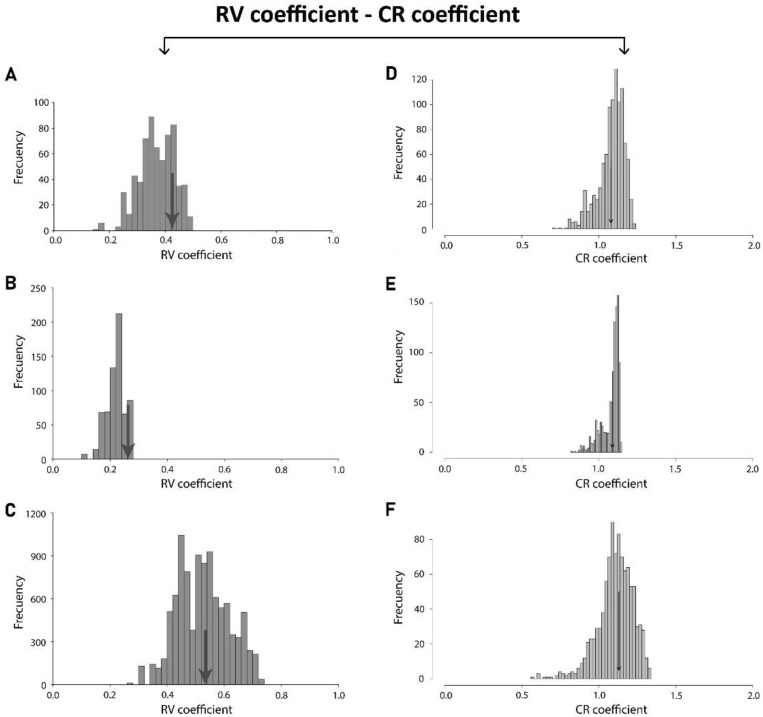
Modularity hypotheses at different integration levels comparing the covariation between anterior and posterior compartments of the wing. (**A**) Static RV coefficient, (**B**) Developmental RV coefficient, (**C**) Evolutionary RV coefficient, (**D**) Static CR coefficient, (**E**) Developmental CR coefficient (**F**) Evolutionary CR coefficient. The arrows indicate the RV and CR coefficient between the anterior and posterior compartments, and the histograms represent the distribution of coefficients for the alternative landmark partitions.

For the modularity tests, the RV coefficient was 0.422, and the arrow representing it was quite on the right portion of the distribution graph rejecting the modularity hypothesis (Figure 4A). The CR test reject the hypothesis of modularity with a CR: 1.0787 *p* -value: 0.394 (Figure 4D). This means that these two parts do not represent different modules. The modularity test across species clearly rejected the proposed anterior and posterior hypothesis.

The regression of wing shape on centroid size pooled by species and sex accounted for only a 4.37% of the variation in shape (*p* < 0.0001), thus indicating that there is a significant but low static allometry on the data.

### 3.2. Developmental Level

The PCA from the covariance matrix of the asymmetric component indicated a moderate developmental integration (Figure 3B, Table 2). The first three dimensions accounted 36.1% of the total variance, the overall levels of integration accounted for 0.03720.

Although the overall strength of association between A/P compartments indicated a weak relation between them (RV: 0.26), the permutation tests indicated a highly significant relationship (*p*-value: < 0.001). The FA covariation pattern between the anterior and posterior compartment of the wings for the first three singular values accounted for most of the covariance of the A/P compartments (about the 77% of the total squared covariance) and therefore, provided a reasonable summary of the covariation pattern.

The same procedure carried out to analyse the overall integration level separated by species, was also carried out for the developmental integration levels. These results showed that the static and the developmental integration levels were relatively similar among all the analysed species.

The modularity test for the fluctuating asymmetry shows a RV value of 0.2 with the arrow at the extreme right part of the distribution graph rejecting the modularity hypothesis (Figure 4B). To compare with CR test this also reject the hypothesis with CR: 0.7827, *p*-value: 0.094 (Figure 4E). This means that these two parts do not represent different developmental modules.

Developmental allometry only accounted for a miniscule 0.86% of the variation in shape, thus indicating there is a minimal size effect at the developmental level.

### 3.3. Evolutionary Level

The analysis of the independent contrasts indicated that there is clear evolutionary integration of the overall wing structure. The eigenvalues of the first three PCs accumulated approximately 71% of shape variation (Figure 3C). The overall level of integration for the evolutionary integration accounted for 0.20443.

The overall strength of the evolutionary association showed a noticeable covariation strength (RV: 0.53) with a highly significant permutation (*p* < 0.001).

The PLS analysis of the independent contrasts, showed that the first two pairs of PLS axes accounted for 75% and 15.1% of the total squared covariance between the anterior and posterior wing compartments.

The shape pattern in the evolutionary covariation showed a difference as compared to the other two levels of integration in PLS1 (Figure 3). However, the relationship between landmark displacements did not show large changes in comparison with the other two levels. This change can be described as a relatively displacement of landmark 12 along the longitudinal vein and the relative displacement of landmarks 8 and 9, thus producing a more oval phenotype.

The modularity test for the independent contrast shows a value of RV: 0.53 with the arrow at the right part of the distribution graph rejecting the modularity hypothesis (Figure 4C). The CR test of modularity rejected the hypothesis with a CR: 1.1317, *p*-value: 0.548 (Figure 4E). This means that these two parts do not represent different evolutionary modules.

The evolutionary allometry only accounted for 8.54% of the variation in shape. This indicates that there is a low evolutionary allometry, with a significant permutation test (*p* -value: 0.0006).

### 3.4. Comparison within Levels

The main wing shape variation within species can be described as a contrast between a narrower and pointed tip against a broader and more rounded wing tip. This variation of the wing tip shape simultaneously affects the distal regions in both the anterior and posterior compartments, thus contributing to the integration between them.

**Figure 5:**
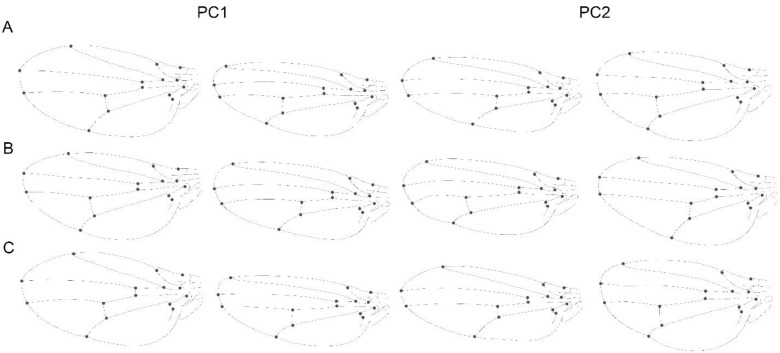
Patterns of wing shape variation associated with the PCs at different levels: (**A**) Static Integration by the covariance matrix of shape pooled by species, (**B**) Developmental integration using the covariance matrix of fluctuating asymmetry and (**C**) Evolutionary integration using the covariance matrix of the independent contrasts of shape. PC1 and PC2 are shown and the figures to the left and right correspond to the shape for each PC score with a magnitude of −0.1 and +0.1, respectively.

**Figure 6:**
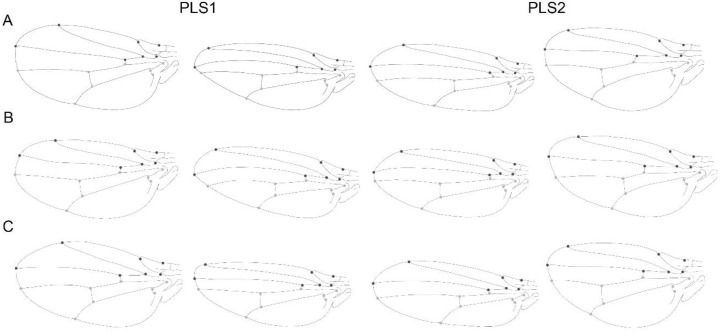
Patterns of wing shape covariation between anterior and posterior compartment associated with the PLS axis at different levels. (**A**) Static Integration by the covariance matrix of shape pooled by species, (**B**) Developmental integration using the covariance matrix of fluctuating asymmetry and (**C**) Evolutionary integration using the covariance matrix of the independent contrasts of shape. PLS1 and PLS2 are shown and the figures to the left and right show the shape for a PLS score with a magnitude of −0.1 and +0.1, respectively.

The pattern of shape variation and covariation as shown by the PCA and PLS analyses were relatively similar at the three levels (Figure 5 and Figure 6, Table 3 and Table 4). The visualization (observed on the extreme positive and negative morphologies) showed how the anterior compartment varies with a relative displacement of landmark 12 and 13, along an expansion of the longitudinal vein (LV), and the posterior compartment the landmark 14 vary generating a more pointed morphology (at the static and evolutionary levels) and slightly rounded to the asymmetry level. The PC2 and PLS2 follow similar patterns but with less abrupt changes. Visualizations of shape changes associated with the results of the PCA, and PLS, showed that the angular comparisons of the vectors directions are quite similar between the levels of integration (Table 3).

A matrix correlation and the angular comparisons between the covariance matrices of the different levels indicate a strong similarity suggesting that the origins are in part similar for the three levels (Table 4). For the size effect across the species, the residuals values for the three levels (i.e., shape scores that are free of the allometric effects) showed that the eigenvalues are slightly lower than in the analysis of uncorrected shape (Table 2).

## 4. Discussion

This study has used a multilevel approach in a comparative context to investigate integration and modularity in *Drosophila*’s wing shape. The main results showed a clear similarity for the analyses of the whole wing structure and for the anterior and posterior parts of the wing. This indicates a substantial degree of trait evolvability, combined with a strong integration. In addition, the modularity hypothesis based on differential developmental origins (i.e., anterior, and posterior compartments) was strongly rejected at the three levels of variation (i.e., RV and CR results).

All the PCAs at the three different levels of variation showed that typically PC1 summarised at least about half of the variation (excepting the static level where the first two PCs explain a similar amount of variance), which is considerably more than any other PC (Figure 3A–C). This pattern indicates that the shape variation in each level is highly concentrated in a single direction, hence confirming a strong integration [5]. Additionally, the shape changes associated with PC1 and PC2 are remarkably consistent across all the three levels of analysis (Figure 3). It is unsurprising for *Drosophila* that the wing shape data contain most of the variation concentrated on the first dimensions. Many geometric morphometric studies showed strong variation associated to the first PC, both for single species [46,48,49], as well as when analysing multiple species [27,29,47,50,51,52,53]. This means that most of the studies have found strong integration in *Drosophila*’s wing. This provides a strong support for the hypothesis that considers integration as a constraint of morphological evolution. Interestingly, Hansen and Houle [54] argue that stabilizing selection cannot account for the stability of *Drosophila* wing shape, and that rather intrinsic constraints are the cause of the observed stasis. Our results indicate that the similarity between the multiple levels of integration could support this wing shape stability hypothesis. Moreover, Houle et al. [29] also corroborates this after found a very low rate of evolution in *Drosophila* wings and similarities among mutational, genetic, and among-species variation. The patterns of shape variation revealed by the PCA and covariation by the PLS are partially concordant with the results of variation and covariation, described by Klingenberg and Zaklan [7] for *Drosophila melanogaster*. Since the PLS axes represent only the covariation between the anterior and posterior compartment of the wing, Klingenberg and Marugán-Lobón [24] explain that since the first few PCs are obtained as those aspects of shape comprising most overall variation, a correspondence between PLS axes and PCs would indicate aspects of integrated evolution. It is noticeable at the Figure 5 and Figure 6 that the relationship between variation and covariation is not identical between the three levels of integration (there is no exact equivalence between the shape changes of the static and developmental PC1 of Figure 5 and among the PLS axes in Figure 6). Nevertheless, they show some level of similarity. This correspondence is also evident when considering the high results obtained from the matrix correlations and the comparison between angles between the covariance matrices for the three levels (Table 4). Similar correspondence between PCs and PLS axes has been found in other studies on diverse organisms, in a maximum of two and three levels of variation [7,13,22,24,55,56,57,58].

During the 80’s and 90’s several developmental studies have suggested that the adult wings should have modules with different developmental origins [59,60,61,62]. However, later it was found that *Drosophila melanogaster* exhibited a correlation between wing compartments that was particularly high, which indicated the integration between these two parts [7,28]. Our results further strengthen this case, as it is now possible to state that irrespective of the species tested or the level of variation (static, developmental and evolutionary) there is a strong covariation between modules across the whole genus. This means that *Drosophila*’s wing seems to have evolved and behaved as an integrated unit.

Since allometry generates shape variation that is totally concentrated in one dimension of the shape space, it is certainly a factor that could contribute to integration trough a complete morphological structure. Consequently, it is highly relevant to consider allometry when carrying out analyses concerning morphological integration and morphological variation [8,22,24,26,52,55,63,64,65,66,67,68]. We assessed the covariance matrices of the residual values of the multivariate regression of shape on centroid size at the different levels, discovering that irrespective of size correction, the values of integration throughout the entire structure and between parts were not influenced. In fact, the relative matrices show striking similarities in integration values (Table 2).

The analysis of FA patterns among and across the species, revealed an overall integration of the wing. The analyses showed a clear correspondence between the spatial patterning of static variation and FA (PCs and PLS, Figure 5 and Figure 6) and with a high and significant matrix correlation between them. There were mostly minor differences in these patterns, and it was primarily the order of the PCs. However, static integration and developmental integration could be commanded by the same developmental processes [3]. It is well known that numerous studies have made such comparisons in a variety of different organisms, showing widely varying results [2,24,69,70,71,72,73,74]. Analyses focused on the wings of *Drosophila*, as well as other insects have tended to show concordance between the patterns of covariation for FA and individual variation. This suggests that direct developmental interaction is important at all levels of covariation [5,55,62,75].

On the other hand, the present results showed that evolutionary integration exhibits a large concentration of variation in one direction during the evolution of the genus, (first dimension of the PCA) (Figure 3A–C). Due to the fact that independent contrasts denote evolutionary change, the covariation between independent contrasts of the shape coordinates for the whole structure and for the anterior and posterior compartment implies an evolutionary integration pattern [24]. The present results indicated that entire wing appears to evolve as a coordinated unit. In addition, the pattern of shape variation and covariation also present similarities with the other two levels of integration, mostly the evolutionary level with the static level, thus provides insights about of the origins of the integration of *Drosophila*’s wing shape. This similarity could suggest that the observed integration patterns could be product of evolutionary processes driven by drift or some other selective constraints [54]. Therefore, our results at the evolutionary level combined with the results obtained for the other levels of variation have been able to help us to understand some of the possible mechanisms responsible for the slight phenotypic variation. When these results are analysed in the light of their similarities with the other two covariance matrices, it is possible to conclude that morphological integration act as the main constraint for wing shape variation, being therefore probably one of the main processes involved in the wing shape diversification of this group.

Nevertheless, common objections to constraint to explain those similarities or morphological stasis, is the notion that quantitative characters are very evolvable because they show plenty amounts of additive genetic variance and new mutational variation which are related with the phenotypic variation. [29,51,61].

Only few studies have been carried out using GM and a multilevel integration approach. Most of them have found correspondences between static and evolutionary integration [22,46,51,63]. However, for other levels of variation several other studies in different taxa have contributed by providing information about the mechanisms responsible for phenotypic variation, such as canalization, plasticity or even developmental instability and their adaptative change [21,22,24,30,58,70,75,76,77,78,79,80].

Therefore, the present results represent the first integration and modularity analyses at multiple levels in the comparative context of the genus *Drosophila*. Our results also can help to understand the different integration origins involved in the generation of the *Drosophila* wing variation. The strong integration and common pattern of variation at multiple levels, suggest a shared mechanism underlying this variation, and raise the question that the evolutionary patterns of variation are perhaps the product of genetic drift acting and it is new evidence of stasis in the *Drosophila* wing shape in the genus [27,54].

## Figures and Tables

**Table 1:** Measurement error Procrustes ANOVA for both *Drosophila*’s wing centroid size and shape, characterised by matching symmetry.

Centroid Size							
Effect	SS	MS	df	F	*p*	Pillai tr.	*p* (param)
Individual	11.478684	0.604141	19	1466.1	<0.0001		
Side	0.001664	0.001664	1	4.04	0.0589		
Ind × Side	0.007829	0.000412	19	3.07	0.0014		
Error 1	0.005366	0.000134	40				
**Shape**							
Effect							
Individual	0.1654094	3.35 × 10^−4^	494	52.68	<0.0001		
Side	0.0001051	4.04 × 10^−6^	26	0.64	0.919		
Ind × Side	0.0031397	6.36 × 10^−6^	494	9.67	<0.0001	13.14	<0.0001
Error 1	0.0006835	6.57 × 10^−7^	1040				

**Table 2:** Principal component analysis between three levels of variation: Static (pooled by species), fluctuating asymmetry, and evolutionary, with their corresponding values after allometry correction (A). The table values are the eigenvalues and percentages of total variance for the first three PCs accounts.

	Eigenvalues			% Total Variance			
Level of Integation	PC1	PC2	PC3	PC1	PC2	PC3	Cumulative
Static	0.00004798	0.00003807	0.00002962	21.103	16.744	13.025	50.872
Developmental	0.00002477	0.00001866	0.00001616	15.019	11.312	9.801	36.132
Evolutionary	0.00023655	0.00010357	0.00006316	41.987	18.383	11.21	71.58
Static (A)	0.00003817	0.00003626	0.00002842	17.94	17.043	13.356	48.339
Developmental (A)	0.00002467	0.00001808	0.00001603	15.09	11.055	9.808	35.953
Evolutionary (A)	0.00020041	0.00009249	0.00005925	39.558	18.256	11.695	69.509

**Table 3:** Angular comparison between the vectors of first three PC’S and PLS’s between different levels of variation.

Static Integration	Angular Value
PC1-PLS1	16.675°
PC2-PLS2	29.974°
PC3-PLS3	28.923°
**Developmental Integration**	
PC1-PLS1	19.868°
PC2-PLS2	61.799°
PC3-PLS3	66.981°
**Evolutionary Integration**	
PC1-PLS1	7.297°
PC2-PLS2	17.697°
PC3-PLS3	27.616°

**Table 4:** Matrix correlation between the different covariance matrices at different levels of variation: A: Static Integration by the covariance matrix of shape pooled by species, B: Developmental integration using the covariance matrix of fluctuating asymmetry and C: Evolutionary integration using the covariance matrix of the independent contrast of shape.

Matrix Correlation/*p*-Value	Developmental Integration	Evolutionary Integration
Static Integration	0.95038357	0.85631863
Developmental Integration	-	0.74279388
**Matrix Correlation/*p*-Value**	**Developmental Integration**	**Evolutionary Integration**
Static Integration	<0.0001	<0.0001
Developmental Integration	-	<0.0001

## Data Availability

Not applicable.

## Supplementary Materials

The following supporting information can be downloaded at: https://www.mdpi.com/article/10.3390/biology11040567/s1; Table S1: Principal component analysis of variation between two levels of variation for the 59 species and one subspecies: individual variation and fluctuating asymmetry. The table values are the eigenvalues and percentages of total variance for the first three PCs accounts. Table S2: Partial least square analysis of the anterior and posterior compartment of the wing shape for the 59 species and one subspecies. The table values are the two first PLS scores and the RV coefficient.
